# Relative likeability and relative popularity as sources of influence in children’s friendships

**DOI:** 10.1371/journal.pone.0283117

**Published:** 2023-05-12

**Authors:** Brett Laursen, Mary Page Leggett-James, Olivia M. Valdes

**Affiliations:** Florida Atlantic University, Boca Raton, Florida, United States of America; University of Valencia: Universitat de Valencia, SPAIN

## Abstract

The present study contrasts two forms of peer status as sources of friend influence: Relative likeability and relative popularity. Participants included 310 children (142 boys, 168 girls), ages 9 to 12, in stable reciprocated friendships. Peer nominations were collected at two time points, 8 to 14 weeks apart. After removing overlapping variance through residualization, partners in each friend dyad were categorized into roles on the basis of relative (to the partner) popularity and relative (to the partner) likeability. Dyadic analyses compared more- and less-liked friends and more- and less-popular friends in terms of their influence over physical aggression, relational aggression, prosocial behavior, and academic achievement. Higher initial relational aggression, prosocial behavior, and academic achievement among more-liked partners predicted greater increases in the same among less-liked partners, but not the reverse. Unexpectedly, physical aggression among less-liked partners predicted increases in physical aggression among more-liked partners. More popular friends did not influence less popular friends on any of these variables, although (also unexpectedly) less-popular friends influenced the academic achievement of more-popular friends. Taken together, the findings suggest that during the pre- and early adolescent years, relative influence within a friendship tends to be apportioned on the basis of likeability, not popularity.

## Introduction

Peers increasingly dominate a child’s social world during the late primary and early middle school years. Friends, in turn, dominate the social world of peers [1]. We know that friends exercise considerable influence over a host of adjustment outcomes [2]. Less clear are the dynamics that drive influence within a friendship. What makes one friend more influential than another? Peer status is often invoked to answer this question [3]. Both likeability (an index of preference) and popularity (an index of dominance) have been tied to attributes that lend themselves to being influential. No prospective study, however, has identified the relative contribution of each. The present longitudinal study compares likeability and popularity as sources of influence within the friendships of 9–12 year old children, describing the degree to which status (relative to the partner) determines the degree to which one friend shapes the other’s prosocial behavior, academic achievement, and relational and physical aggression across a 2–3-month period.

We begin with a few definitions. *Peers* are agemates; in the context of the present study peers refers to classmates. *Friends* are preferred peer affiliates; in the context of the present study, best friends refer to classmates who reciprocally nominate one another as top ranked friends. We operationalize *influence* in terms of change: A child alters the way he or she behaves in response to experiences with others [4]. Peer influence is often assumed to be deleterious, but it need not be so. Positive influence is just as likely as negative influence [5]. Influence can be seen in groups, through interactions with peers. Influence can also be seen in dyads, through interactions with a friend. We focus here on friend dyads, consistent with research suggesting that best friends wield considerable, unique influence over adjustment, particularly during the pre- and early adolescent years when unsupervised peer groups are limited in their scope and reach [2].

The existence of peer influence is well-established. Less is known, however, about who influences whom. Two forms of peer status have been implicated in friend influence. *Likeability* (also known as social preference) is an affective measure that reflects the degree to which a child is liked or disliked by peers. It is typically measured by classmate ratings (e.g., How much do you like Dora?) or by subtracting like-least nominations received (Who do you dislike?) from like-most nominations received (e.g., Who do you like?). *Popularity* is a reputation-based measure that reflects perceptions of dominance and leadership in the group. It is typically measured through peer nominations (Who is popular?). We know that adolescents conform to high status peers [e.g., 6] but it is not clear which form of status is more important. Both popularity and likeability have been linked to conformity, but different influence mechanisms are postulated for each.

Well-liked children have more friends than their lesser-liked counterparts and more options for making new friends (in that more classmates claim to like them) [7]. Within a dyad, the better-liked friend is assumed to be influential because they have an arsenal of persuasive, noncoercive interpersonal tools to draw upon. The less-liked friend is assumed to be susceptible to influence because they desire to preserve amity and are wary about antagonizing a rewarding affiliate who may prove difficult to replace [8]. Support for these claims can be found in studies of friend dyads indicating that relatively higher accepted friends influence their relatively lower accepted partners (but not the reverse) in domains such as school achievement [9] and alcohol misuse and delinquent behavior [10]. Social network studies indicate that absolute levels of liking are also tied to influence over school grades [11] and alcohol intoxication [12].

Popular children are leaders whose attitudes and actions are closely monitored by peers. Their popularity is derived from the strategic use of aggression and/or prosocial behavior [13]. Within a dyad, the more-popular friend is assumed to be more influential, through a calibrated application of power, pressure, and rewards. The less-popular friend is assumed to be susceptible to influence, in part because they are more submissive and in part because they value the protection and reflected glory that popular friends provide [2]. Support for these claims is found in studies that report positive associations within adolescent friendship networks between absolute popularity and school grades [11], aggressive behavior [14] and prosocial behavior [15]. Less is known about friend dyads, but one study found that the relatively more popular friend influenced the substance use and risk-taking preferences of their relatively less popular friend, but not the reverse [16].

During the late primary school and early middle school years, likeability and popularity are independent, but overlapping (meta-analytic *r* = .47) constructs [17]. It is not surprising, therefore, that children label well-liked and popular classmates as influential [18]. For the same reason, however, separate studies of likeability and popularity cannot offer definitive conclusions about the unique contributions of each to friend influence. Which raises the question: Does likeability or popularity drive influence within a friendship?

One study anticipated the focus of our research in that it addressed the relative importance of popularity and likeability in friend influence. The findings were not particularly conclusive. Results from a longitudinal social network study of adolescent attitudes towards immigrants [19] revealed that changes in intergroup attitudes were related to absolute levels of friend likeability but not to absolute levels friend popularity; the more a child was liked by others (regardless of the status of a friend) the more influence the child exerted over a friend. Separate analyses were conducted with each peer status variable, so their relative effects could not be determined.

The present study is unique in that it employs a longitudinal dyadic framework to examine the degree to which higher status friends influence lower status partners across a 2-3-month period within a school year. Designed for correlated, dyadic data, the Actor-Partner Interdependence Model [20] partitions variance shared across partners on the same variable from variance that uniquely describes associations within partners and between partners. Modifications for longitudinal data estimate the influence that the relatively higher and relatively lower status friend have over one another [21]. We focus on friend influence during late primary and early middle school years, when peer conformity is at its peak.

Two competing hypotheses will be considered. One asserts the primacy of likeability, holding that better-liked friends influence the behavior of lesser-liked partners on the grounds that nonconformity poses an acute risk to friendship participation for relatively less-liked friends, who have fewer close affiliates and fewer options for replacing them. The other asserts the primacy of popularity, holding that more-popular friends influence the behavior of less-popular partners on the grounds that popular children possess traits that are synonymous with influence, such as dominance, leadership, and a willingness and ability to deploy social control strategies to achieve aims. To this end, partners within each friend dyad were categorized according to their relative popularity and relative likeability, using residualized scores to isolate the influence. Likeability was regressed on popularity to distinguish the relatively better-liked friend from the relatively lesser-liked partner; popularity was regressed on likeability to distinguish the relatively more-popular friend from the relatively less-popular partner.

Some have hypothesized that boys are more susceptible to peer influence than girls [22] and previous studies suggest gender differences in concurrent associations between peer status and adjustment [3], so gender was examined as a potential moderator. Age-group differences have been identified in patterns of friend similarity [e.g., 23] and in associations between likeability and popularity [17], so we also examined age as a potential moderator.

## Method

### Participants

The final sample included 310 children (142 boys, 168 girls) attending two public primary and middle schools required to represent public school students in the state of Florida in terms of ethnicity and family income. Primary school students were in the 4^th^ and 5^th^ grade; middle school students were in the 6^th^ grade. Primary school students retained the same classmates across the school day. Middle school students attended classes with a rotating subset of students from the same pod (i.e., two classes that met jointly once a day during homerooms, typically the first period of the day, for attendance, announcements, and other nonacademic activities).

Two cohorts of students participated. *Cohort 1* consisted of 66 4^th^ graders (*M* = 9.52 years, *SD* = 0.53), 64 5^th^ graders (*M* = 10.56 years, *SD* = 0.54) and 48 6^th^ graders (*M* = 11.42 years, *SD* = 0.55) during the 2013–14 academic year. *Cohort 2* consisted of 34 3^rd^ graders (*M* = 8.16 years, *SD* = 0.44), 46 4^th^ graders (*M* = 9.26 years, *SD* = 0.61) and 52 5^th^ graders (*M* = 10.25 years, *SD* = 0.40) during the 2015–16 academic year. Of this total, 38.1% were European American, 17.3% were African American, 28.0% were Hispanic American, and the remainder identified mixed or other backgrounds.

### Procedure

Written parent consent and written child assent were required for participation. The project was approved by school officials and the Florida Atlantic University IRB (702335–8). In 35 of 36 classrooms, participation rates averaged 76.8% (*SD* = 8.10); one class with participation rate below 60% was excluded from the analyses. Participants completed questionnaires at two time points, 8 or 14 weeks apart. Questionnaires were administered by trained research assistants during regular class hours.

### Measures

#### Stable best friendships

At both time points, participants identified and rank-ordered an unlimited number of friends (“Who are your friends?”) from a roster of same-gender classmates. Stable reciprocated best friends nominated one another as a top-ranked friend at both time points. Most children participated in more than one stable reciprocated friendship (*M* = 4.04, *SD =* 1.60). Friendship length was measured by child reports of the academic year the friendship began.

#### Peer nominations

At both time points, participants completed a peer assessment questionnaire [24] consisting of a class roster on which they circled the names of those who best fit each description. Unlimited same-gender nominations were permitted. Rosters were limited to same-gender participants to limit gender stereotyping [25] and (in the 6th grade) to minimize fatigue and error arising from the need to parse lengthy class lists. Because each informant in a peer nomination procedure is treated as a separate indicator, single-item nominations are generally considered reliable, although nominations for readily observable traits, such as popularity and prosocial behavior, tend to be more reliable than those for inner states or preferences, such as likeability and relational aggression [26]. For each variable, the number of nominations a child received was standardized to adjust for class size using a regression-based procedure that corrects for bias tied to the number of nominators [27].

Two indices of status were assayed. *Popularity* was measured with the item: “*Someone who is popular*.” A residual popularity score was computed by regressing popularity onto likeability, thus identifying variance unique to popularity. *Likeability* was measured differently in the two cohorts. In cohort 1, a roster-rating procedure was employed [28]. Participants rated all same-gender classmates (“*How much do you like this person*”) on a scale ranging from 1 (*do not like this person*) to 5 *(like this person very much*). In cohort 2, likeability was measured using a standard sociometric nomination procedure. Working from a class roster, participants were permitted an unlimited number of liked-most (“*like to spend time with*”) and liked-least (“*don’t like to spend time with*”) nominations. Likeability was calculated as the difference between the number of liked-most nominations received and the number of liked least nominations received [29]. The two strategies tend to yield similar assessments of likeability [30]. Likeability scores were standardized within cohort. A residual likeability score was computed by regressing likeability onto popularity, thus identifying variance unique to likeability.

*Relational aggression* was measured with the item: “*Someone who talks bad about others behind their back*.*” Physical aggression* was measured with the item *“Someone who hits*, *pushes or shoves people*.*” Academic achievement* was measured with the item: “*Someone who does well in school*.*” Prosocial behavior* was measured (in cohort 1 only) with the item: *“Someone who makes sure everyone is treated equally*.*”*

#### Plan of analysis

Distinguishable dyad, longitudinal Actor-Partner Interdependence Model (APIM) analyses were conducted in a structural equation modeling framework using Mplus v8.2 [31] with MLR estimation. APIM analyses partition variance shared across partners on the same variable from variance that uniquely describes associations within partners (intraindividual) and between partners (interindividual). To better separate interindividual change from intraindividual change, we model latent-difference scores in a longitudinal framework [see 32, for an illustration]. APIM analyses cannot readily account for bias that may arise from nested data, but concerns can be reduced with the Mplus COMPLEX function [33], which corrects standard errors based on the degree of nonindependence of observations arising from sample clustering (e.g., friendships nested within classrooms).

There was no attrition, so there was no wave-level missingness (i.e., all participants had complete data at each time point). Prosocial behavior was not collected in cohort 2 and was, by definition, missing completely at random. The same pattern of statistically significant results emerged when prosocial behavior analyses were restricted to cohort 1 participants.

Of the 723 students who completed surveys at both time points, 678 reported at least one reciprocated friendship. Of this total, 32 were excluded because none of their reciprocated friendships were stable across both time points. Participants were restricted to a single friendship, with preference given to the highest ranked partner. An additional 186 students were eliminated because all of their friends were involved in higher ranked reciprocated friendships. Another 80 students were excluded because their likeability or popularity scores were identical to those of their friends, meaning that partners could not be distinguished (see below). A final 70 students were eliminated because they were involved in dyads in which more- and less-liked friends or more- and less-popular friends switched roles from Time 1 to Time 2. T-tests revealed no greater than chance differences on any study or demographic variable between the final sample and (a) students eliminated because their friendships were not stable (*d*_studyvar_ = .03 to .11, *d*_demographicvar_ = .02 to .17), and (b) students eliminated because their friends were members of other friendship dyads, (*d*_studyvar_ = .04 to .14, *d*_demographicvar_ = .04 to .12). Reciprocated top-ranked friends represented 80% of dyads included in the analyses; one or both friends were ranked 2^nd^ in 12.3% of dyads; lower ranked friends were included in 7.8% of dyads.

Each friend in each dyad was classified as either relatively more likeable (*M* = 3.05, *SD* = 2.06) or relatively less likeable (*M* = 1.84, *SD* = 2.60). Separately, each friend in each dyad was classified as either relatively more popular (*M* = 3.98 *SD* = 3.80) or relatively less popular (*M* = 2.08, *SD* = 2.93). Chi-square tests of distinguishability [20] confirmed the need for distinguishable dyad APIM analyses: residual likeability, χ^2^(6) = 60.85 to 314.24 *p <* .01; residual popularity, χ^2^(6) = 16.25 to 326.22, *p*≤.01. There was no systematic overlap between being designated the more-popular friend within a dyad and being designated the better-liked friend, χ^2^(1) = 0.32, *p* = .57. The better-liked friend was just as apt to be the less-popular friend (52%) as the more-popular friend (48%).

**Fig 1:**
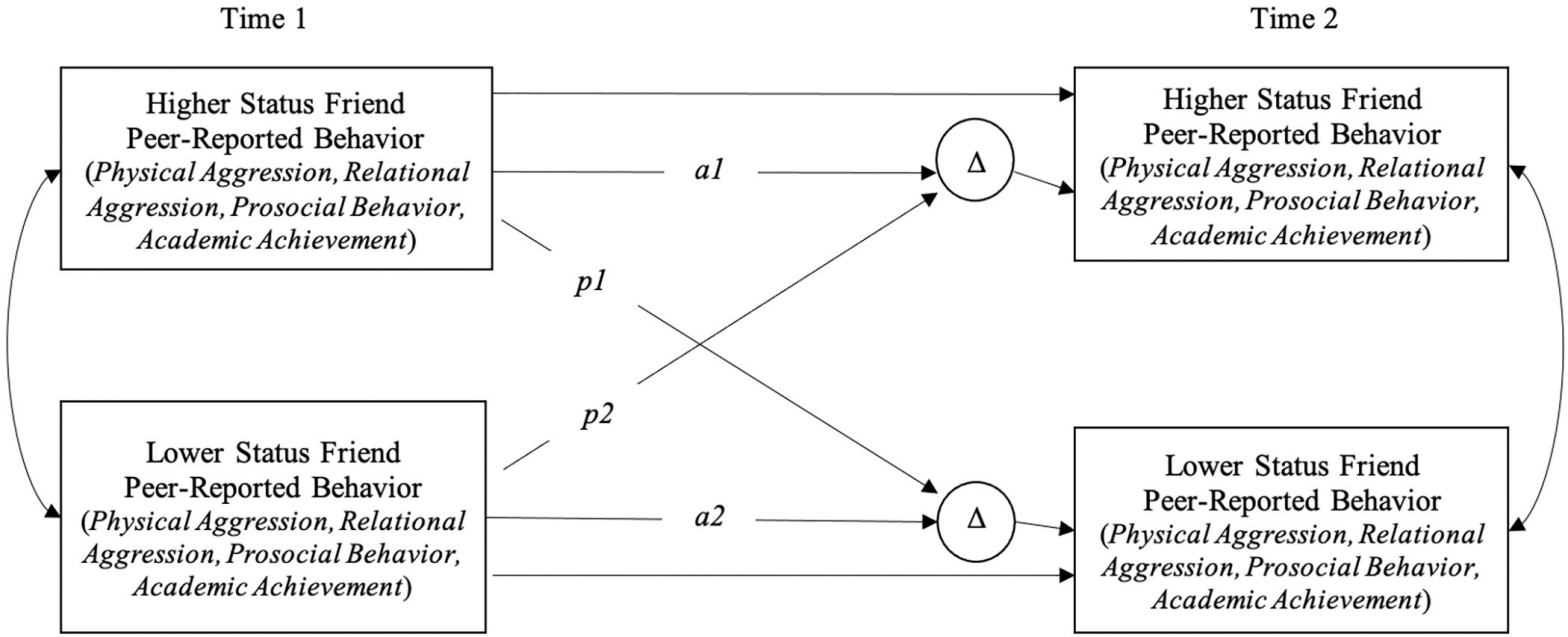
Longitudinal latent change actor-partner interdependence model for distinguishable dyads: Measurement model. *Note*. Stability (actor) paths = *a1* and *a2*. Influence (partner) paths = *p1* and *p2*. Separate analyses were conducted for friends distinguished on relative residual likeability (Higher Status Friend = More-Liked Partner, Lower Status Friend = Less-Liked Partner) and for friends distinguished on relative residual popularity (Higher Status Friend = More-Popular Partner, Lower Status Friend = Less-Popular Partner).

Fig 1 depicts the distinguishable dyad longitudinal APIM analytic model. Friend influence is indicated by intraindividual partner path (*p1* and *p2*). A significant *p1* path indicates that the initial behavior of the higher status friend predicted changes in the behavior of the lower status friend. A significant *p2* partner path indicates that the behavior of the lower status friend predicted changes in the behavior of the higher status friend. Separate sets of analyses were conducted for friends distinguished by residual likeability and for friends distinguished by residual popularity. In each case, influence was assessed in four domains: relational aggression; physical aggression; academic achievement; and prosocial behavior.

A series of supplemental analyses were conducted. First, to ensure that effects were unique to friends, we reran the analyses on a comparison group of randomly paired same-grade, same-gender nonfriend participants, neither of whom nominated the other as a friend at either time point.

Second, potential confounds were separately added to the model as Time 1 correlates. Friendship length and rank were included as covariates to mitigate differences in relationship closeness. The average scores of all other nominated friends were included as covariates, to rule out the possibility that partner influence was driven by the larger network of peer affiliates. Friend difference scores on each behavioral attribute (i.e., relational aggression, physical aggression, academic achievement, and prosocial behavior) were included as covariates, to disentangle the effects of initial behavioral dissimilarity from those of relative peer status. Age was also entered as a covariate, to confirm the absence of developmental differences. Finally, each friend’s peer status score was entered as a covariate, to ensure that influence effects did not vary as a function of absolute levels of acceptance and popularity. In each case, the same pattern of statistically significant results emerged.

Third, multiple-group analyses were conducted to identify moderators of friend influence. With one exception (described in the results), there were no statistically significant differences as a function of cohort, *Wald*(1) = .22–2.28, *p* = .13-.64 or gender, *Wald*(1) = 3.07–4.20, *p* = .07-.08, suggesting that influence did not differ as a function of these variables. Previous studies report that more-accepted friends exert greater influence over less-accepted friends if they also report higher levels of the attribute that is the subject of influence [9]. To test this hypothesis, we divided dyads into two groups, according to the relative level of the behavioral attribute of the higher accepted friend (e.g., more-accepted friends with relatively higher levels of prosocial behavior and more-accepted friends with relatively lower-levels of prosocial behavior). Similar analyses examined whether differences of the behavioral attribute of the less-accepted friend were related to influence (e.g., less-accepted friends with relatively higher levels of prosocial behavior and less-accepted friends with relatively lower-levels of prosocial behavior). There were no statistically significant differences, *Wald*(1) = .62–3.46, *p* = .06-.43.

Monte Carlo power analyses [34] were conducted to determine the whether the number of dyads available per group was adequate (i.e., 80%) to detect statistically significant effects. There was adequate power to detect large effects for contrasts involving moderators of influence (.88-.99, *M*_*power*_ = .90), but much less power to detect medium effects (.24-.81, *M*_*power*_ = .58) and small effects (.09-.17, *M*_*power*_ = .13).

## Results

### Preliminary analyses

Correlations and ANOVAs were conducted on one randomly selected member of each friend dyad. Table 1 presents means, standard deviations, and intercorrelations between study variables. All autocorrelations were statistically significant (*p* < .05). At both time points, likeability was (a) positively correlated with popularity, prosocial behavior, and academic achievement, and (b) negatively correlated with physical aggression (Time 2 only). At both time points, popularity was positively correlated with prosocial behavior and academic achievement. Data from friends were not independent, a precondition for longitudinal APIM analysis: Friends resembled each other on all study variables (intraclass *r* = .439-.796, *p* < .01).

**Table 1:** Concurrent correlations, autocorrelations, means, and standard deviations.

Variable	1	2	3	4	5	6	*M*	(*SD*)
1. Likeability	.56** [.44, .66]	.14 [-.02, .29]	-.38** [-.51, -.24]	-.35** [-.48, -.20]	.34** [.20, .48]	.16* [-.01, .31]	0.53	(0.82)
2. Popularity	.14 [-.02, .29]	.91** [.88, .94]	-.02 [-.17, .13]	-.04 [-.19, .12]	.26** [.11, .40]	.46** [.33, .57]	3.19	(3.56)
3. Relational Aggression	-.49** [-.60, -.36]	-.04 [-.20, .11]	.74** [.66, .80]	.49** [.36, .60]	-.17* [-.31, -.01]	-.19* [-.33, -.03]	0.57	(1.11)
4. Physical Aggression	-.34* [-.47, -.19]	-.04 [-.18, .13]	.56** [.50, .66]	.77** [.70, .83]	-.21** [-.35, -.05]	-.25** [-.39, -.09]	0.44	(.87)
5. Prosocial Behavior	.33** [.19, .47]	.29** [.13, .42]	-.21** [-.36, -.06]	-.17* [-.32, -.02]	.79** [,72, .84]	.58** [.46, .67]	3.33	(2.34)
6. Academic Achievement	.19* [.03, .34]	.45** [.32, .57]	-.17* [-.32, -.02]	-.18* [-.32, -.02]	.51** [.39, .62]	.92** [.89, .94]	4.79	(3.90)
*M*	0.46	3.43	0.70	0.48	3.22	5.39		
*(SD)*	(0.81)	(3.65)	(1.28)	(0.97)	(2.30)	(4.53)		

*Note*. *N =* 155 students representing one randomly selected member from each friend dyad. The APIM analyses were conducted with residual likability and residual popularity scores, but standardized scores of likeability and raw scores of other variables are presented here for ease of interpretation. Time 1 results are presented above the diagonal. Time 2 results are presented below the diagonal. Autocorrelations are presented on the diagonal. *p < .05, **p < .01.

Separate 2 (gender) x 2 (cohort) ANOVAs were conducted with time as the repeated measure. Relational aggression, physical aggression, prosocial behavior, and academic achievement were the dependent variables. There were statistically significant main effects for gender on relational aggression, *F*(1, 147) = 7.86, *p* = .006, and physical aggression, *F*(1, 147) = 27.91, *p* < .01. Compared to girls, boys had higher scores on relational aggression (*d* = 0.36) and physical aggression (*d* = 0.68). There was a statistically significant effect for time on academic achievement, *F*(1, 153) = 26.14, *p* < .01, *d* = 0.14, and on relational aggression, *F*(1, 153) = 6.06, *p* = .015, *d* = 0.16, with both increasing from Time 1 to Time 2. There were no cohort differences.

### APIM analyses for friends distinguished on the basis of residual likeability

The left side of Table 2 summarizes the findings for analyses in which friends were distinguished on the basis of residual likeability.

**Table 2:** Influence within friend dyads: Results from longitudinal actor-partner interdependence models.

	Residual Likeability				Residual Popularity			
Variable	*a1*	*a2*	*p1*	*p2*	*a1*	*a2*	*p1*	*p2*
Relational Aggression	-.33** [-0.55, -0.11]	-.27** [-0.45, -0.08]	.23* [0.02, 0.44]	.16 [-0.08, 0.40]	-.25* [-0.45, -0.06]	-.10 [-0.10, 0.18]	.11 [-0.12, 0.34]	.04 [-0.10, 0.18]
Physical Aggression	-.50** [-0.74, -0.26]	-.20 [-0.43, 0.03]	-.09 [-0.36, 0.18]	.24* [0.00, 0.48]	-.24** [-0.41, -0.07]	-.32** [-0.57, -0.08]	.01 [-0.18, 0.20]	.03 [-0.16, 0.21]
Prosocial Behavior	-.50** [-0.63, -0.37]	-.40** [-0.59, -0.20]	.16* [0.02, 0.28]	.11 [-0.15, 0.37]	-.34** [-0.48, -0.20]	-.47** [-0.64, -0.30]	.14 [-0.04, 0.31]	-.02 [-0.21, 0.17]
Academic Achievement	-.03 [-0.25, 0.19]	-.30** [-0.49, -0.11]	.43** [0.22, 0.63]	.08 [-0.13, 0.30]	-.21* [-0.41, -0.01]	-.06 [-0.21, 0.10]	.16 [-0.04, 0.36]	.29* [0.07, 0.51]

*Note*. *N* = 155 friend dyads. Standardized beta weights presented. The measurement model (Fig 1) illustrates each path. *a1* = stability of high-status friend’s behavior from Time 1 to Time 2, *a2* = stability of low status friend’s behavior from Time 1 to Time 2, *p1* = influence of high-status friend on low status friend from Time 1 to Time 2, *p2* = influence of low status friend on high status friend from Time 1 to Time 2. Confidence intervals [95%] given in brackets. *p < .05, **p < .01.

### Relational aggression

The more-liked friend’s relational aggression was positively associated with changes in the less-liked friend’s relational aggression from Time 1 to Time 2. In contrast, the less-liked friend’s relational aggression did not influence the subsequent relational aggression of the more-liked friend.

### Physical aggression

The more-liked friend’s physical aggression was not associated with changes in the less-liked friend’s physical aggression from Time 1 to Time 2. Contrary to hypotheses, the less-liked friend’s physical aggression was positively associated with changes in the more-liked friend’s subsequent physical aggression.

### Prosocial behavior

The more-liked friend’s prosocial behavior was positively associated with changes in the less-liked friend’s prosocial behavior from Time 1 to Time 2. In contrast, the less-liked friend’s prosocial behavior did not influence the subsequent prosocial behavior of the more-liked friend.

### Academic achievement

The more-liked friend’s academic achievement was positively associated with changes in the less-liked friend’s academic achievement from Time 1 to Time 2. In contrast, the less-liked friend’s academic achievement did not influence the subsequent academic achievement of the more-liked friend.

### Supplemental analyses

S1 Table presents results from analyses conducted on a comparison group of random pairs of same-grade, same-gender participants. There were no statistically significant influence paths for relational aggression (β = -.06 to -.15, *p* = .23 to .42), physical aggression (β = .03 to .14, *p* = .12 to .64), prosocial behavior (β = -.03 to .04, *p* = .65 to .75), and academic achievement (β = -.03 to -.01, *p* = .50 to .71).

### APIM analyses for friends distinguished on the basis of residual popularity

The right side of Table 2 summarizes the findings for analyses in which friends were distinguished on the basis of residual popularity.

### Relational aggression

The more-popular friend’s relational aggression was not associated with changes in the less-popular friend’s relational aggression from Time 1 to Time 2. Similarly, the less-popular friend’s relational aggression did not influence the more-popular friend’s subsequent relational aggression.

### Physical aggression

The more-popular friend’s physical aggression was not associated with changes in the less-popular friend’s physical aggression from Time 1 to Time 2. Similarly, the less-popular friend’s physical aggression did not influence the more-popular friend’s physical aggression.

### Prosocial behavior

The more-popular friend’s prosocial behavior was not associated with changes in the less-popular friend’s prosocial behavior from Time 1 to Time 2. Similarly, the less-popular friend’s prosocial behavior did not influence the more-popular friend’s subsequent prosocial behavior.

### Academic achievement

The more-popular friend’s academic achievement was not associated with changes in the less-popular friend’s academic achievement from Time 1 to Time 2. Contrary to hypotheses, the less-popular friend’s academic achievement was positively associated with changes in the more-popular friends’ subsequent academic achievement.

### Supplemental analyses

S1 Table presents results from analyses conducted on a comparison group of random pairs of same-grade, same-gender participants. There were no statistically significant influence paths for relational aggression (β = -.06 to -.02, *p* = .22 to .87), physical aggression (β = -.02 to .08, *p* = .36 to .70), prosocial behavior (β = -.03 to .01, *p* = .67 to .99), and academic achievement (β = -.03 to .01, *p* = .55 to .76). Contrary to hypotheses, the less-popular friend’s academic achievement was positively associated with changes in the more-popular friend’s subsequent academic achievement. Follow up gender contrasts, *Wald*(1) = 6.20, *p* = .01, indicated that the effect was statistically significant for boys (*β* = .22, *p* < .001) but not for girls (*β* = .03, *p* = 64).

## Discussion

We sought to identify sources of influence within children’s friendships, tracking changes in physical and relational aggression, academic achievement, and prosocial behavior across a 2-3-month period. Previous research indicates that higher status friends hold sway over lower status friends [e.g., 6], but until now it was not clear if these effects are a product of differences in likeability, differences in popularity, or both. Across most outcomes, when partners differed in terms of likeability, better-liked friends influenced their less-liked counterparts and not the reverse. Equally consistent were the findings for popularity: Within friend dyads, more-popular partners did not influence less-popular partners. We conclude that when it comes the importance of different markers of status, influence within the friendships of 9–12 year old children is apportioned on the basis of likeability, not popularity.

Influence may flow from characteristics of the agent, characteristics of the target, or both. Although we can say with some certainty that better liked partners influence lesser liked partners, our study design does not permit us to distinguish variance attributable to being influential from that attributable to being susceptible to influence. Better-liked partners may be influential because of attributes associated with likeability. Being well liked is positively associated with adaptive social skills [35], which suggests that better-liked friends may be good at persuading others to accede, perhaps through the use of prosocial arguments [36]. Being well liked is also associated with a host of desirable attributes, including accomplishment, which may prompt others to emulate modeled behaviors in the hopes of attaining similar success, and attractiveness, which may elicit compliance with the goal of maintaining proximity and basking in reflected glory [37]. Finally, well-liked children are perceived to be fun [38]; maintaining the rewards of an enjoyable companion may be a powerful enticement to conformity.

Equally likely, however, is the prospect that lesser-liked partners are particularly susceptible to influence. Some children are not well liked because they are shy [39], others are not well liked because they are anxious [40]; both may give rise to conformity as a strategy to reduce social pressure. Perhaps even more important, low accepted youth have fewer options for friends than their higher accepted partners, which may provide a strong incentive to avoid conflict (from nonconformity) that could prove disruptive [41]. Children with few options for friends may conclude that it is better to concede to a friend than to lose a friend. Perceived pressures to conform to norms may also persuade adolescents to adopt the behaviors of higher status others, who tend to establish and enforce group norms [42, 43]. Finally, a confluence of factors may contribute to individual differences in susceptible to influence from peers, including uneven neurological maturation and unfinished identity exploration [8].

Other longitudinal studies suggest that relatively more popular friends influence their relatively less popular counterparts [16]. Why did we fail to find effects for popularity? We can only speculate. Perhaps most obviously, acceptance is a measure that reflects the sum of an individual’s relations with each member of the group (how many classmates like the child) whereas popularity is a measure of the individual’s standing in the group’s hierarchy (how many classmates place the child in a dominant position). Studies that look only at popularity may be capitalizing on variance shared with likeability. Influence at the level of the group does not necessarily translate into influence at the level of the dyad. Children may associate different forms of influence with acceptance and popularity [44]. Personal influence (“I look to this person for how to act”) is tied to acceptance, whereas reputational influence (e.g., “others look to this person for how to act”) is tied to popularity. In dyads, children may be influenced by better liked peers, whereas in groups, children may be influenced by popular peers. Further, previous studies have focused on students older than those in the current study. Some have suggested that as the salience of the peer group grows across middle adolescence, so too should influence arising from popularity [22]. Caution is also warranted in the interpretation of some unanticipated findings. The lesser-liked friend influenced the better-liked friend’s physical aggression, and the less-popular friend influenced the more-popular friend’s academic achievement. We are hard pressed to explain these results and will not engage in post-hoc speculation about them.

Acceptance and popularity are not mutually exclusive. In a few instances, a child’s best friend is also the most popular person in the peer group, but these instances reflect a relatively small portion of all friend dyads. It is also possible that findings may have been strongest in instances where the relatively more accepted friend was also the relatively more popular partner. We lacked the power to adequately test additive effects. Note that the analyses only address relative status within a friend dyad (i.e., which friend has more status). They do not speak to whether children with higher levels of status within a classroom hold more influence over classmates, a question better addressed with social network analyses.

Our study is novel. It the first to contrast relative likeability and relative popularity in a longitudinal framework, a strategy necessary to disentangle similarity arising from selection from similarity arising from influence. Our residualization strategy ensured that friend differences were specific to likeability or acceptance. Confidence in the findings was bolstered by the fact that similar patterns of influence were not found among randomly selected peers; the findings were unique to friends. Finally, we considered several potential confounds and none altered the pattern of results, suggesting that the findings were not a product of the length or importance of the relationship nor did the findings differ as a function of absolute levels of the outcome variable or the magnitude of friend status differences. Of course, we cannot rule out the possibility that friend influence is tied to influence from the entire network of peers, but controls for the average ratings of all nominated peers help to mitigate this concern.

Several limitations are worth noting. First, small sample sizes limited power in moderator analyses, tempering conclusions about null findings for gender and initial levels of the outcome variable. Second, our reliance on classmate reports (as opposed to friend reports) means that descriptions of individual behaviors were not tied to relationships. To the extent that the two are inconsistent, classmate reports should make it harder (not easier) to identify partner-specific associations. Third, our sample included a mix of longstanding and recently established friendships. Previous studies suggest that pressure for increased similarity is greatest during and shortly after friendship formation; the later phases of a friendship appear to be focused more on maintaining similarity, rather than increasing it [21]. As a consequence, the results likely underestimated the magnitude of friend influence. Finally, reputation rosters were limited to same-gender participants. The consequences of this practice are not fully understood; increases in error variance arising from the inclusion of fewer raters may be offset by decreases in error that may accompany other-gender stereotyping and the need to review extensive lists of potential nominees.

Among 9–12 year old friends who differ on peer status, influence tends to be a function of relative likeability, not relative popularity: Better liked-friends influence less-liked partners, but more popular friends do not influence less popular partners. Scholars would be well advised to focus their efforts on the apportionment of influence within friend dyads to relative acceptance, recognizing that popularity is more apt to be a source of influence at the group level. Of course, this is not the final word on the topic. Influence is undoubtedly tied to different factors in different friendships. The fact that many friends do not differ on likeability or popularity, suggests the need to look beyond peer status, to factors such as relationship satisfaction [45], which has also been tied to differences in friend influence. A change in tactics also may be required. Person-oriented research strategies [46] would seem to be an appropriate alternative avenue for determining which attributes are sources of influence in which relationships.

## Supporting information

S1 Table Influence within nonfriend dyads: Results from longitudinal actor-partner interdependence models. *Note*. *N* = 155 nonfriend dyads. Standardized beta weights presented. The measurement model (Fig 1) illustrates each path. *a1* = stability of one non-friend’s behavior from Time 1 to Time 2, *a2* = stability of one non-friend’s behavior from Time 1 to Time 2, *p1* = influence of a non-friend on another non-friend from Time 1 to Time 2, *p2* = influence of a non-friend on another non-friend from Time 1 to Time 2. Confidence intervals [95%] given in brackets. *p < .05, **p < .01.
(DOCX)

S1 Data (CSV)
