# Supplementary material for: Relative likeability and relative popularity as sources of influence in children’s friendships
Source: PLoS One. 2023 May 12;18(5):e0283117. doi: 10.1371/journal.pone.0283117 (PMC10180626; doi:10.1371/journal.pone.0283117)
Supplement: S1 Table — Note. N = 155 nonfriend dyads. Standardized beta weights presented. The measurement model (Fig 1) illustrates each path. a1 = stability of one non-friend’s behavior from Time 1 to Time 2, a2 = stability of one non-friend’s behavior from Time 1 to Time 2, p1 = influence of a non-friend on another non-friend from Time 1 to Time 2, p2 = influence of a non-friend on another non-friend from Time 1 to Time 2. Confidence intervals [95%] given in brackets. *p < .05, **p < .01. (DOCX) [file pone.0283117.s001.docx]

**Supporting Information**

**S1 Table. Influence Within Nonfriend Dyads: Results from Longitudinal Actor-Partner Interdependence Models.**

|  | Residual Likeability | | | | | | |  | Residual Popularity | | | | | | |  |
| --- | --- | --- | --- | --- | --- | --- | --- | --- | --- | --- | --- | --- | --- | --- | --- | --- |
| Variable | *a1* | | *a2* | | *p1* | | *p2* |  | *a1* | *a2* | | *p1* | | *p2* | |  |
| Relational  Aggression | .62**  [.42, .82] | .78**  [.66, .90] | | .15  [-.05, .35] | | -.06  [-.18, .06] | |  | .76**  [.60, .93] | | .82**  [.73, .92] | | -.02  [-.21, .17] | | -.06  [-.13, .02] | |
| Physical  Aggression | .73**  [.60, .85] | .76**  [.61, .91] | | .14  [-.01, .28] | | .03  [-.08, .20] | |  | .76**  [.64, .88] | | .82**  [.69, .95] | | .08  [-.07, .23] | | -.02  [-.12, .07] | |
| Prosocial  Behavior | .39**  [.16, .61] | .72**  [.62, .82] | | .04  [-.11, .19] | | -.03  [-.16, .11] | |  | .53**  [.33, .73] | | .71**  [.61, .82] | | .01  [-.14, .14] | | -.03  [-.16, .10] | |
| Academic Achievement | .91**  [.88, .94] | .87**  [.83, .92] | | -.03  [-.10, .04] | | -.01  [-.08, .05] | |  | .90**  [.87, .93] | | .86**  [.80, .92] | | -.03  [-.10, .05] | | .01  [-.06, .08] | |

*Note. N*=155 nonfriend dyads*.* Standardized beta weights presented. The measurement model (Figure 1) illustrates each path. *a1*=stability of one non-friend’s behavior from Time 1 to Time 2, *a2*=stability of one non-friend’s behavior from Time 1 to Time 2, *p1*=influence of a non-friend on another non-friend from Time 1 to Time 2*, p2*=influence of a non-friend on another non-friend from Time 1 to Time 2*.* Confidence intervals [95%] given in brackets. **p*<.05, ***p*<.01.
